# Complete Genome Sequence of a Colistin-Resistant Klebsiella pneumoniae Isolate from Houseflies (Musca domestica) in a Trash Disposal Truck in the United States

**DOI:** 10.1128/MRA.00257-20

**Published:** 2020-05-28

**Authors:** Anwar Kalalah, Anil Poudel, Jiansen Gong, Li Chen, Yi Yang, Chengming Wang

**Affiliations:** a Department of Pathobiology, Auburn University College of Veterinary Medicine, Auburn, Alabama, USA; b Poultry Institute, Chinese Academy of Agricultural Sciences, Yangzhou, Jiangsu, China; c Yangzhou University College of Veterinary Medicine, Yangzhou, Jiangsu, China; University of Maryland School of Medicine

## Abstract

A Klebsiella pneumoniae strain isolated from houseflies in a trash disposal truck in the United States was resistant to colistin, a last-resort drug for treating infections caused by multidrug-resistant Gram-negative bacteria. Complete genome sequencing resulted in a total genome size of 5,337,408 bp for this isolate with a plasmid of 224,442 bp.

## ANNOUNCEMENT

Klebsiella pneumoniae is an opportunistic pathogen and a leading cause of nosocomial infections (1). The emergence of K. pneumoniae resistance to colistin, a last-resort antimicrobial used to treat carbapenem-resistant *Enterobacteriaceae*, has led to fear of pan-drug resistance.

Houseflies have been linked to various bacterial infections as a mechanical vector, but their role in the dissemination of antimicrobial resistance, particularly colistin resistance, remains poorly understood (2–5). Here, we report the complete genome sequence of a colistin-resistant K. pneumoniae strain isolated from houseflies trapped in a trash disposal truck in Auburn, Alabama.

Colistin selective medium CHROMagar COL-APSE (CHROMagar, Paris, France) was used for the isolation of colistin-resistant *Enterobacteriaceae* from fly homogenates at 37°C for 18 h. A mucoid, metallic blue colony suggestive of coliform from Fly ID AU-87 was selected and grown in lysogeny broth supplemented with 3.5 μg/ml colistin sulfate (Sigma-Aldrich, St. Louis, MO) at 37°C for 18 h. A volume of 400 μl from the cultured isolate was used for extraction of genomic DNA with the High Pure PCR template preparation kit (Roche Diagnostic, Indianapolis, IN) according to the manufacturer’s instructions. The isolate was characterized as K. pneumoniae (isolate Kp8701) based on PCR amplification of the full-length 16S rRNA gene using primers 27F (5′-AGAGTTTGATCCTGGCTCAG-3′) and 1492R (5′-GGTTACCTTGTTACGACTT-3′) as previously described, (6) followed by Sanger sequencing (Elim Biopharmaceuticals, Inc., San Francisco, CA). A BLAST search of the obtained sequence was conducted using the NCBI Nucleotide Basic Local Alignment Tool (BLASTn) database.

The single-molecule real-time (SMRT) sequencing library was constructed with the SMRTbell prep kit 1.0 (Pacific Biosciences, Menlo Park, CA) and performed on the PacBio Sequel platform (Oebiotech, Shanghai, China). Reads with low quality were removed with SMRT Analysis 2.3.0 (Pacific Biosciences), and the filtered reads were assembled to generate one contig without gaps using FALCON 1.1 (7). The corrected subreads were used as an input for the single-pass read accuracy improver software SPRAI 0.9.9.23 (8) to maximize the continuity of the assembled contig. Genes were predicted and annotated using the NCBI Prokaryotic Genome Annotation Pipeline (PGAP) (9, 10). Default parameters were used for all software.

SMRT sequencing of Kp8701 resulted in three data sets with roughly 3.65 Gbp total bases available for *de novo* assembly. There were 397,836 total reads with a minimum contig length of 51 bp and a maximum length of 70,234 bp (average length, 9,162 bp). Assuming an average genome size of 5 Mbp for K. pneumoniae, the total bases obtained from SMRT sequencing theoretically had 730-fold coverage of the genome.

The *de novo* assembly of the genome is composed of a single circular chromosome of 5,337,408 bp with a GC content of 57.42% and a circular plasmid (pKp8701) of 224,442 bp with a GC content of 52.37%. There was a total of 5,569 genes predicted in the genome of Kp8701, consisting of 5,319 protein-coding genes, 131 RNA-coding genes, and 119 pseudogenes. The RNA-coding genes included 90 sets of tRNA genes encoding all 21 amino acids, 25 rRNA genes (9 sets for 5S rRNA, 8 sets for 16S rRNA, and 8 sets for 23S rRNA), and 16 noncoding RNA (ncRNA) genes.

### Data availability.

The raw sequencing reads were deposited in the Sequence Read Archive (SRA) under accession number SRR11212804 (BioProject number PRJNA609348). The *de novo* genome assemblies of the K. pneumoniae Kp8701 chromosome and plasmid were deposited in GenBank under accession numbers CP049604 and CP049605, respectively.
